# Long-Term Cultivation and Metagenomics Reveal Ecophysiology of Previously Uncultivated Thermophiles Involved in Biogeochemical Nitrogen Cycle

**DOI:** 10.1264/jsme2.ME17165

**Published:** 2018-03-29

**Authors:** Shingo Kato, Sanae Sakai, Miho Hirai, Eiji Tasumi, Manabu Nishizawa, Katsuhiko Suzuki, Ken Takai

**Affiliations:** 1 Ore Genesis Research Unit, Project Team for Development of New-generation Research Protocol for Submarine Resources, Japan Agency for Marine-Earth Science and Technology (JAMSTEC) Yokosuka, Kanagawa, 237–0061 Japan; 2 Research and Development Center for Submarine Resources, JAMSTEC Yokosuka, Kanagawa, 237–0061 Japan; 3 Department of Subsurface Geobiological Analysis and Research, JAMSTEC Yokosuka, Kanagawa, 237–0061 Japan; 4 Research and Development Center for Marine Biosciences, JAMSTEC Yokosuka, Kanagawa, 237–0061 Japan

**Keywords:** thermophiles, metagenomics, long-term continuous cultivation, nitrogen cycle, subsurface geothermal groundwater

## Abstract

Many thermophiles thriving in a natural high-temperature environment remain uncultivated, and their ecophysiological functions in the biogeochemical cycle remain unclear. In the present study, we performed long-term continuous cultivation at 65°C and 70°C using a microbial mat sample, collected from a subsurface geothermal stream, as the inoculum, and reconstructed the whole genome of the maintained populations using metagenomics. Some metagenome-assembled genomes (MAGs), affiliated into phylum-level bacterial and archaeal clades without cultivated representatives, contained genes involved in nitrogen metabolism including nitrification and denitrification. Our results show genetic components and their potential interactions for the biogeochemical nitrogen cycle in a subsurface geothermal environment.

Phylogenetically diverse, uncultivated thermophiles are present in natural high-temperature environments, such as terrestrial hot springs and deep-sea hydrothermal vents, as suggested by 16S rRNA gene analyses (3, 16, 32). Culture-independent single-cell genomics and metagenomics are powerful techniques and have become standards in microbial ecology (7, 13, 23, 31). Previous studies using single-cell genomics and metagenomics revealed the metagenome-assembled genomes (MAGs) or single amplified genomes (SAGs) of uncultivated thermophiles, and suggested their metabolic potential (4, 27, 30, 34). However, their physiological functions and ecological roles remain unclear because of the absence of cultivated representatives or the availability of partial genome sequences only. One of the research approaches to overcome this difficulty is a genomic analysis based on temperature-controlled laboratory cultivation, which has provided direct evidence for growth temperatures and metabolism (10, 11).

Nitrogen is one of the key elements for life in anabolism and catabolism as well as for the biogeochemical cycle. Uncultivated thermophiles detected in a high-temperature environment potentially play significant roles in the biogeochemical nitrogen cycle, as indicated by their metabolic potential (4–6, 27). In order to obtain a better understanding of the mechanisms by which as well as what thermophilic microbial populations contribute to the nitrogen cycle in the natural environment, we collected a microbial mat sample in an ammonia-rich geothermal groundwater stream in Japan and performed continuous cultivation at 65°C and 70°C using flow-through bioreactors with the mat sample as the inoculum as previously reported (25). At the original sampling site, discharged groundwater has constantly shown a high temperature (69–72°C), circumneutral pH (6.1–6.4), low salinity (0.1%), and high concentrations of NH_4_^+^ (>100 μM) and Fe^2+^ (>70 μM) (14, 17, 24, 33). Previous studies demonstrated the presence of uncultivated microorganisms affiliated with *Candidatus* (*Ca.*) “*Nitrosocaldus*”, “Aigarchaeota”, and “Acetothermia” in the stream and inoculated mat sample (14, 24–27, 34). The MAGs of “Aigarchaeota” and “Acetothermia” in the stream have already been reported (27, 34). Although no MAGs/SAGs of “*Nitrosocaldus*” have been described to date, “*Nitrosocaldus*” spp. have predominated in microbial communities as shown by a 16S rRNA gene analysis (24, 26). Geochemical measurements and a nitrogen isotopic analysis of mat samples have provided evidence for the microbiological oxidation of ammonia and nitrite (24). Furthermore, the oxidation of ammonia to nitrite by “*Nitrosocaldus*” spp. at 70°C was demonstrated using bioreactors for more than 2 years (25). These findings have suggested that the nitrogen cycle, such as nitrification, is driven by these uncultivated microorganisms in this high-temperature environment.

We herein demonstrated the metabolic potential of maintained populations in high-temperature bioreactors, which operated for more than 4 years, as revealed by metagenomics. Details of the experimental procedures employed are described in Supplementary Information. In brief, DNA was extracted from samples in a bioreactor (Reactor-65), which operated at 65°C for 349 d after the inoculation, and in another bioreactor (Reactor-70A), which operated at 70°C for 45 and 1,579 d after the inoculation. On day 159, a subsample from Reactor-70A was inoculated into a new bioreactor (Reactor-70B), which operated at 70°C, in order to assess the reproducibility of cultivation, and a sample was obtained from Reactor-70B 1,420 d after the re-initiation of cultivation. DNA extraction was performed in duplicate, except for Reactor-70A on day 45. Extracted DNA from seven samples was applied to shotgun metagenomic sequencing as previously described (12). All reads were cleaned up (Table S1) and co-assembled. This resulted in 26,561 contigs (≥1 kbp) with an N_50_ value of 10,355 bp. Binning using longer contigs (≥2.5 kbp) and manual curation resulted in a total of 41 MAGs (Fig. S1). The MIMAG (Minimum Information about a Metagenome-Assembled Genome) developed by the Genomic Standards Consortium (8) as a standard for reporting MAGs, such as estimates of genome completeness and contamination, was summarized in Table S2. Phylogenetic trees were constructed for 16S rRNA gene sequences (Fig. S2) and for the concatenated protein sequences of 43 single-copy marker genes (Fig. 1A).

These MAGs were assigned to 19 bacterial clades including “Calescamantes”, “Fervidibacteria”, and “Parcubacteria” (30), “Dadabacteria” (15), “Fischerbacteria” (1), and GAL15 defined as a phylum-level clade in the Silva database (29) without cultivated representatives, and two archaeal clades including “Aigarchaeota” and *Thaumarchaeota* at the phylum level (see Supplementary Information for details on the taxonomic affiliations of MAGs). Thaumarchaeotic MAGs were classified into two lineages—”*Nitrosocaldus*” and a deeper lineage most closely related (16S rRNA gene similarity, 90.3%) to the environmental clone Papm3A43 recovered from deep-sea hydrothermal fluid (18). Based on cultivation temperatures and periods, all MAG-derived cultivates were thermophilic or thermo-tolerant.

In order to assess the relative abundance of these MAG populations in the bioreactor, read coverages for MAGs were displayed as a heat-map figure (Fig. 1B; Table S2). Several MAGs in “Aigarchaeota”, “*Nitrosocaldus*”, *Acidobacteria*, and GAL15 were relatively abundant in all samples from the bioreactors at 65°C and 70°C over time. Some MAGs in *Armatimonadetes*, “Fischerbacteria”, “Calescamantes”, *Chloroflexi*, and “Aigarchaeota” were less abundant at 65°C, but were relatively abundant at 70°C, which suggests that they were adapted to the higher temperature.

In order to clarify the ecological roles of the maintained populations in the nitrogen cycle, such as nitrification catalyzed by ammonia monooxygenase (Amo), hydroxylamine oxidoreductase (Hao), and nitrite oxidoreductase (Nxr), assimilatory and dissimilatory nitrate reduction by nitrate and nitrite reductases (Nap/Nar/Nas/Nir), denitrification by nitric oxide reductase (Nor) and nitrous oxide reductase (Nos), and nitrogen fixation by nitrogenase (Nif), we focused on genes for the key subunits of the above enzymes (Fig. 2).

The *amoABC* genes were found in the “*Nitrosocaldus*” MAGs (HR04 and HR05), but not in any of the other MAGs or unbinned contigs. These *amo* genes have also been detected in enrichment cultures of “*Nitrosocaldus yellowstonii*” (10); however, its entire genome has not been reported. Although the oxidation of NH_3_ to NO_2_^−^ by the “*Nitrosocaldus*”-related populations in our bioreactors has been demonstrated previously (25) and in the present study (Fig. S3), the enzymes that catalyze NH_2_OH to NO_2_^−^ in archaeal ammonia-oxidizers have yet to be identified (20). Since a *hao* gene was detected in an unbinned contig, we were unable to exclude the possibility that NH_2_OH oxidation was catalyzed by an undefined cultivate. The *amo* genes were not found in Papm3A43-related MAG (HR06), and were also absent in the previously reported MAGs and SAGs of deeper-branching thaumarchaeotic lineages than “*Nitrosocaldus*” (4, 22, 30). As suggested previously (4, 22), the HR06-derived archaeon may not be an ammonia oxidizer.

The *narGH*/*nxrAB* and *nirK* genes were found in Papm3A43-related MAGs, in addition to some MAGs of “Aigarchaeota” and several bacterial MAGs. We performed a phylogenetic analysis of the subunit NarH/NxrB (Fig. S4), which has been used in an Nar/Nxr analysis (28), indicating that the NarH/NxrB sequences obtained were distantly related to previously identified NxrB proteins. Thus, a phylogenetic analysis was not sufficient to identify which gene products were realistically involved in microbial NO_2_^−^ oxidation. Nevertheless, NO_3_^−^ production was observed in the bioreactors (up to 150 μM in the effluent) operated at 70°C for more than 4 years (Fig. S3), indicating that some genes for Nar/Nxr were involved in NO_2_^−^ oxidation to NO_3_^−^. Nearly half of the MAGs (19 out of 41) including “Aigarchaeota”, “Fischerbacteria”, and “Dadabacteria” contained *nosZ*. Thus, they may produce N_2_ from N_2_O, which may be produced abiotically from NH_2_OH and NO (21) or biotically from NO via NorBC.

Our results provide insights into previously unrecognized physiological and metabolic properties, particularly in the nitrogen cycle, of thermophilic microbial populations in high-temperature bioreactors, some of which represent phylum-level clades without cultivated representatives. *nosZ* has not been found in the MAGs/SAGs of “Aigarchaeota” reported to date. However, HR02, one of the “Aigarchaeota” MAGs found in this study, contained the gene. Although the contribution to the nitrogen cycle of deep-branching thaumarchaeotic lineages including the MAG named Fn1 (22) is unknown, the HR06-derived archaeon with *nar*/*nxr* may be involved in nitrite oxidation or nitrate reduction. No MAGs/SAGs of “Dadabacteria”, “Fischerbacteria”, or GAL15 have been obtained from any geothermal environment to date. Thus, HR11, HR31, HR32, and HR37 are the first thermophilic MAGs in these groups, and contain several genes involved in the nitrogen cycle, similar to previously reported MAGs from a nongeothermal environment (1, 15). Several MAGs/SAGs of “Calescamantes” and “Fervidibacteria” have been reported from geothermal environments, and contain genes involved in the nitrogen cycle, such as *narG*, *nirS*, and *nosZ* (6, 30), which is consistent with our results.

In addition to nitrogen metabolism, other potential metabolic functions of MAGs, such as carbon fixation and energy acquisition, are summarized in Table S3 and Supporting Information. In the bioreactors, the energy source for sustaining the microcosm was limited to NH_3_ added to media at a final concentration of approximately 200 μM (12; Supporting Information). The ammonia-oxidizing archaeal populations derived from the abundant “*Nitrosocaldus*” MAG (HR04) may be primary producers in the microcosm as suggested previously (12). NO_2_^−^ produced from the oxidation of NH_3_ may be used by nitrite-oxidizing autotrophs potentially derived from some of the MAGs containing *nxr*/*nar* genes. However, the carbon fixation pathways present have yet to be identified. Unexpectedly, Fe(II), which was in media at a final concentration of approximately 100 μM to simulate the chemical composition of geothermal groundwater at the sampling site, may be another potential energy source for the population. The “Fischerbacteria” and Chlorobi MAGs (HR11 and HR21) contained a homolog for Cyc2 (Fig. S5), an outer membrane *c*-type cytochrome that may catalyze Fe(II) oxidation in several iron oxidizers, such as *Acidithiobacillus* spp., *Ferriphaselus* spp., *Gallionella* spp., and *Mariprofundus* spp. (2, 9, 19). Moreover, a near-complete gene set including *rbcSL* in the Calvin cycle for carbon fixation was found in HR11. These results imply that the bacterium derived from the “Fischerbacteria” MAG is potentially an iron-oxidizing autotroph and has a role as a primary producer in the microcosm.

The present study revealed the unique physiological functions and ecological roles of diverse, previously uncultivated thermophiles in the nitrogen cycle in a subsurface geothermal environment. These results provide important information for the isolation of novel MAG-derived thermophiles, and will contribute to the characterization and elucidation of their ecophysiologies. Further cultivation-based metagenomics for a number of high-temperature environments with different pH, salinities, redox potentials, and energy sources may reveal uncultivated thermophiles and unidentified thermophilic microbial ecosystems.

## Supplementary Material



## Figures and Tables

**Fig. 1:**
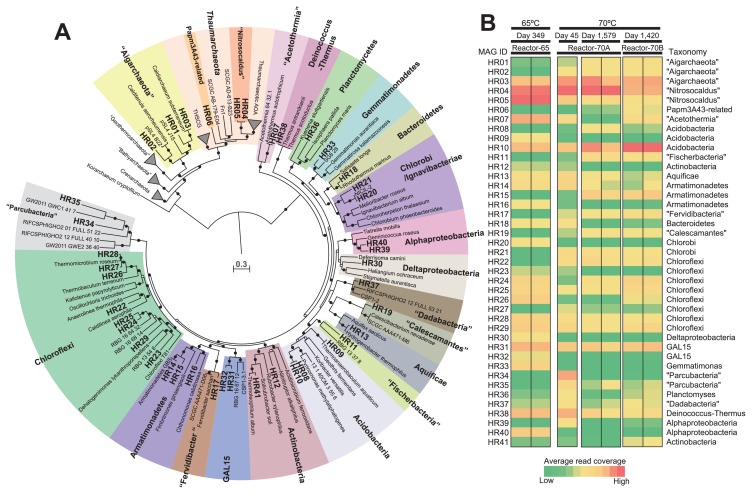
Phylogeny and relative abundance of cultivates based on the analysis of MAGs. (A) Maximum likelihood tree of concatenated amino acid sequences of 43 conserved single-copy marker proteins. The MAGs obtained in this study are shown in bold. The tree was rooted at the midpoint between *Archaea* and *Bacteria*. Filled and open circles at branches indicate more than 70% and 50–70% of bootstrap values (1,000 replicates), respectively. The scale bar represents 0.3 amino acid substitutions per sequence position. (B) Heat-map representing the relative abundance of MAGs in each metagenome (a total of seven) based on average read coverages.

**Fig. 2:**
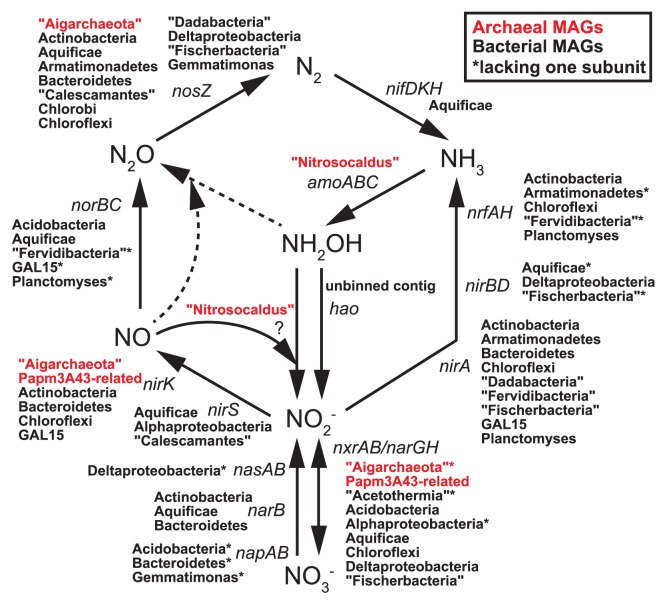
Potential contribution of MAG-derived cultivates in the biogeochemical nitrogen cycle. Taxonomic names for the archaeal and bacterial MAGs are colored in red and black, respectively. Solid and dashed arrows indicate enzymatic and abiotic reactions, respectively. Asterisks indicate MAGs lacking a gene for one of the key subunits of the enzymes (see Table S3 for details).
