# Supplementary material for: Long-Term Cultivation and Metagenomics Reveal Ecophysiology of Previously Uncultivated Thermophiles Involved in Biogeochemical Nitrogen Cycle
Source: Microbes Environ. 2018 Mar 29;33(1):107–10. doi: 10.1264/jsme2.ME17165 (PMC5877337; doi:10.1264/jsme2.ME17165)
Supplement: Supplementary file 1 [file 33_107_s1.pdf]

Supplementary information

Supplementary methods

Reactor operation

Details of the collection of the inoculum sample for cultivation, operation of the continuous-flow bioreactor, and the chemical composition of the culture medium used in this study has already been reported (16). In this study, we operated two bioreactors, one at 65°C (Reactor-65) and the other at 70°C (Reactor-70A). The pH of the media was adjusted to 6.8 at the beginning; however, pH changed to 8.0 during cultivation. Glass containers of the bioreactors were filled with porous ceramics (Aero ring; Kyorin, Hyogo, Japan) and pumice (approximately 3–5 mm in diameter) as carrier materials for microbial cells (16). Reactor-65 was operated for 349 days, and operation of Reactor-70A is still being continued. At Day 159 of Reactor-70A operation, a subsample of the carrier material was collected from the glass container, and inoculated into an additional bioreactor operated at 70°C (Reactor-70B) to assess reproducibility of the cultivation. Thus, Day 159 for Reactor-70A corresponded to Day 1 for Reactor-70B. The samples of the influent and effluent media for chemical analysis were collected over time, filtrated with 0.2 μm pore size membrane filters, and stored at -20°C. Samples of carrier material for DNA analysis were collected at Day 349 for Reactor-65 in duplicate, at Day 45 for Reactor-70A once, and at Day 1,579 for Reactor-70A and Day 1,420 for Reactor-70B in duplicate, and stored at -20°C.

Chemical analysis

Nitrite and nitrate ion concentrations in the influent and effluent samples were directly measured using an ion exchange HPLC system: column, IC-Pac A25S (150 × 4 mm, Thermo Fisher Scientific, MA, USA); eluent, 20 mM sodium chloride solution at 1 $mL \cdot min^{-1}$; column temperature, 40°C; and detector, UV/VIS detector (GL-7451, GL Science Inc., Tokyo, Japan) at 210 nm. To determine the total ammonia concentration in the influent and effluent samples, the HPLC flow-through fractions were reacted with an OPA reagent, 20 mM o-phthalaldehyde (methanol solution)/200 mM sodium borate containing 2 mM sodium sulfite (pH 9.2) = 1:4 (v/v) at 50°C. The fluorescence (Ex = 320 nm, Em = 390 nm) of OPA derivative was measured using a fluorescence

detector (RF-20A, Shimadzu, Kyoto, Japan) connected with the HPLC system. The pH was measured using a handy type pH meter (HORIBA LAQUA twin pH B-712).

DNA extraction and sequencing

Genomic DNA was extracted from the seven samples (100–200 mg) individually using a FastDNA SPIN Kit for Soil (MP Biomedicals, Santa Ana, California, USA). Extracted DNA was used for shotgun library construction using a KAPA Hyper Prep Kit (for Illumina) (KAPA Biosystems, Wilmington, MA, USA). The metagenomic libraries were analyzed on an Illumina MiSeq platform as previously reported (7).

Sequence analysis

Adapter sequences were removed from raw reads, low-quality ends of the reads were trimmed, and reads shorter than 100 bp were removed using CLC Genomics Workbench version 9.5.3 (QIAGEN Aarhus A/S) with the default setting. All filtered reads (a total of 8.4 Gbp) from the seven samples were co-assembled using SPAdes version 3.9.0 (2) with the parameter "-k 33, 55, 77, 99," and resulted in 26,561 contigs (1,000 bp or longer) with N50 of 10,355 bp. For each sample, filtered reads were mapped onto contigs using BBmap version 37.36 (https://sourceforge.net/projects/bbmap/). Contigs (2,500 bp or longer) and mapping data were used for binning with MetaBAT version 2.11.1 based on tetranucleotide frequency and read coverage (11). The binned contigs were visualized in terms of G+C content vs. coverage, or coverage for one sample vs. coverage for another sample using gbtools version 2.5.6 (22) and manually curated. The binned and curated contigs were treated as metagenome-assembled genomes (MAGs). Completeness and contamination levels for the MAGs were calculated by CheckM version 1.0.7 (18) based on counting 145 and 104 conserved single-copy genes (CSCGs) for the domains *Archaea* and *Bacteria*.

The MAGs were annotated using Prokka version 1.12 (23). Metabolic pathways were predicted using the Kyoto Encyclopedia of Genes and Genomes (KEGG) pathway tool (17). The module completion ratio (MCR) in each functional module defined by KEGG was calculated using MAPLE version 2.3.0 (27). Functional properties of proteins encoded by protein-coding genes (CDSs) were predicted using InterProScan version 5.24-63.0 (29).

Amino acid sequences were aligned using MUSCLE version 3.8.31 (5). The alignments were trimmed using TrimAl version 1.2re59 with the "-automated1" option (4). For the "genome tree," the 43 single-copy marker genes selected in the previous report (18) were extracted, and their amino acid sequences were aligned using MUSCLE individually. The alignments were concatenated and trimmed using TrimAl as described above. Maximum likelihood (ML) trees were constructed using RAxML version 8.2.9 with the PROTGAMMALG model (24). The nucleotide sequences of 16S rRNA genes were aligned using SINA version 1.2.11 (19) on the Silva website (20). The alignment was trimmed using TrimAl with the "-automated1" option. An ML tree was constructed using RAxML with the GTRGAMMA model. Bootstrap values were computed with 1000 replicates. ARB version 5.5 (15) and iTOL version 3 (13) were used for tree visualization.

Supplementary results and discussion

Reproducibility of sampling and cultivation

To assess the reproducibility of the metagenomic results of sampling from a reactor, we collected two samples from each reactor at each time point – Day 349 from Reactor-65, Day 1,579 from Reactor-70A, and Day 1,420 from Reactor-70B (i.e., on the same date to Day 1,579 for Reactor-70A). However, only one sample was collected from Reactor-70A on Day 45. Overall, the relative abundance trend of the MAGs for each reactor were similar between the two samples, but there was a slight difference for some MAGs. For instance, a difference between HR03 and HR10 in Reactor-70A at Day 1,579, was observed. Comparison of the relative abundance between Reactor-70A at Day 1,579 and Reactor-70B at Day 1,420 confirmed the reproducibility of the cultivation of representative members at 70°C.

Ammonia, nitrite, nitrate, and pH of the influent and effluent of the culture media

The pH slightly decreased from 8.0 of the influent to 7.7 of the effluent for all bioreactors over time. This was probably due to the following reaction by ammonia-oxidizing archaea (AOA): $NH_3 + 1.5\ O_2 = NO_2^- + H_2O + H^+$. Indeed, for all the bioreactors over time, NH_3 consumption and NO_2 production were observed (Fig. S4). The degree of NH_3 consumption and NO_2 production was greater in Reactor-70A at Days 1,569 and 1,651 than that in Reactor-70B at Days 1,410 and 1,502. This result was consistent with the higher relative abundance of "*Nitrosocaldus*" MAGs of AOA in

Reactor-70A at Day 1,579 than that in Reactor-70B at Day 1,420 (Fig. 1B). Nitrate concentration increased in Reactor-70A at Days 1,569 and 1,651 and in Reactor-70B at Days 1,410 and 1,502, but the increase in concentration varied (4–150 μM). This may reflect the difference in the activity of microorganisms related to the nitrate production and consumption.

Taxonomic affiliation of the MAGs

Archaeal MAGs

One of the MAGs affiliated in "Aigarchaeota," HR03, was almost identical to the *Ca*. "*Caldiarchaeum subterraneum*" based on the genome tree (Fig. 1A). HR03 and "*C. subterraneum*" were classified into subgroup G9, which is one of the subgroups in "Aigarchaeota" defined previously (6). The 16S rRNA gene of another "Aigarchaeota" MAG, HR02, was closely related to the environmental clone gxs0811 that has been assigned in the subgroup G2 (6). Although the affiliation of another "Aigarchaeota" MAG (HR01) in the "Aigarchaeota" subgroups was unclear, this MAG was more closely related to HR03 than to HR02.

Based on the 16S rRNA gene similarity, the "*Nitrosocaldus*" MAGs (HR04 and HR05) were closely related (96.1%) to *Ca*. "*Nitrosocaldus yellowstonii*" (Fig. S2). Because the whole genome of "*N. yellowstonii*" has not been reported, we could not add it to the genome tree.

The genome and 16S rRNA gene trees indicated that the Papm3A43-related MAG (HR06) was located between the clade of AOA including "*Nitrosocaldus*" and the Terrestrial Hot Spring Crenarchaeotic Group (THSCG) (25) including a MAG of DS1 recovered in a terrestrial hot spring in the USA (3). In the genome tree, the HR06 was related to several MAGs or single-cell genomes (SAGs), such as Fn1 from a peat bog (14) and SAG_AB-179-E04 from a terrestrial hot spring (21).

Bacterial MAGs

Several bacterial MAGs were affiliated in the phylum-level clades without cultivated representatives including *Ca*. "Dadabacteria" (9), "Acetothermia," "Calescamantes," "Fervidibacteria" and "Parcubacteria" (21), "Fischerbacteria" (1), and GAL15 (defined as a phylum-level clade in the Silva database release 128 based on the 16S rRNA gene). In particular, the MAG of HR07 in the "Acetothermia" was almost identical to *Ca*. "*Acetothermum autotrophicum*" (26). The MAGs of GAL15, HR31 and HR32, were closely related to

RBG_16_67_12 (1) and CSP1-3 (9). Although CSP1-3 was affiliated in Armatimonadetes in a previous report (9), this group should be treated as a phylum-level clade based on our phylogenetic analyses (Fig. 1A and Fig. S2) and also on the classification in the SILVA database release 128.

Some MAGs were affiliated in class- or order-level clades without cultivated representatives, which have been defined as uncultured clone groups based on 16S rRNA gene analysis. The MAGs of HR20 and HR21 in the phylum Chlorobi, which has been recently refined based on genome-based analysis (8), were classified in the OPB56 clade (10). Based on the 16S rRNA gene tree in the phylum Chloroflexi, the MAGs of HR24 and HR25 and the MAG of HR29 were classified in the clades of S085 and TK10, respectively, which were defined in the Silva database release 128.

Several MAGs (7 out of 41) were closely related to the genome of cultivated species including HR27 and HR28 to *Thermomicrobium* spp. of Chloroflexi, HR41 to *Thermoleophilum* spp. of Actinobacteria, HR09 to *Thermoanaerobaculum* spp. of Acidbacteria, HR13 to *Hydrogenobacter* spp. of Aquificae, HR18 to *Rhodothermus* spp., and HR38 to *Thermus* spp. of Deinococcus-Thermus. All of those cultivated species are thermophiles.

Metabolic potential of MAG-derived cultivates

Central carbon metabolism

MAGs in *Ca*. “Fischerbacteria*”* (HR11) and Alphaproteobacteria (HR40) contained genes involved in the Calvin cycle with high MCR values, including the small and large subunits of ribulose-1,5-bisphosphate carboxylase/oxygenase (RbcS and RbcL). This indicated that these members could autotrophically grow. A gene encoding citryl-CoA lyase (Ccl), one of the key enzymes for the reductive TCA cycle that is commonly observed in autotrophic members in Aquificae, was detected in the MAG in Aquificae (HR13). No genes of the key enzymes, such as carbon-monoxide dehydrogenase (Coo), for the reductive acetyl-CoA pathway (known as the Wood–Ljungdahl pathway) were detected in the MAGs. Previously reported genomes of AOA, such as *Nitrosopumilus maritimus* (28) and *Nitrososphaera viennensis* (12), have a 3-hydroxypropionate/4-hydroxybutyrate (3-HP/4-HB) pathway for carbon fixation. Contrary to expectation, we could not identify a 3-HP/4-HB pathway nor another carbon fixation pathway in the “*Nitrosocaldus*” MAGs (HR04 and HR05) maybe because of the low values of genome completeness. However, the “*Nitrosocaldus*” MAGs probably represent an autotrophic AOA

because of the high relative abundance of these MAGs at both 65°C and 70°C throughout the duration of the experiment (Fig. 1B), the medium content containing ammonia as the main energy source, and the presence of *amoABC* genes.

Most of the MAGs contained genes for the Embden-Meyerhof (EM) pathway involved in glycolysis/gluconeogenesis (glucose to pyruvate, or pyruvate to glucose), for pyruvate oxidation to acetyl-CoA, and for the TCA cycle involved in generation of NADH and quinol from acetyl-CoA with high MCR values (>70%). In addition, some MAGs contained genes for the Entner–Doudoroff (ED) pathway involved in glycolysis with high MCR values.

Genes for beta oxidation involved in generation of NADH, $FADH_2$, and acetyl-CoA from fatty acids were found in several MAGs including HR32 in GAL15, HR33 in Gemmatimonas, and HR37 in "Dadabacteria" with 100% MCR. These members likely grow using fatty acids as energy and carbon sources.

Most MAGs contained genes for alcohol dehydrogenase. The MAGs in Armatimonadetes, "Fervidibacteria," and Bacteroidetes contained genes for lactate dehydrogenase. Genes for acetate production were also found in some MAGs. These members potentially perform fermentation.

Sulfur metabolism

Genes for sulfate adenylyltransferase (Sat) and sulfite reductase (Sir) involved in assimilatory sulfate reduction were detected in some MAGs. In particular, a complete gene set (the MCR value, 100%) for assimilatory sulfate reduction was found in four MAGs including HR10 in Acidobacteria, HR17 in "Fervidibacteria," HR19 in "Calescamantes," and HR29 in Chloroflexi. A complete gene set for thiosulfate oxidation system (Sox) was found only in one MAG, HR39 in Alphaproteobacteria. No genes for dissimilatory sulfite reductase (Dsr) or adenosine phosphosulfate reductase (Apr) involved in dissimilatory sulfate reduction were detected in the MAGs.

Aerobic respiration and ATP synthesis

Most MAGs contained genes for terminal oxygen reductases, such as cytochrome *c* oxidase (Cox), *cbb_3*-type cytochrome *c* oxidase (cbb3-Cox), and cytochrome *d* ubiquinol oxidase (Cyd), which suggests that they are aerobes. This is consistent with the aerobic conditions of the cultivation. Genes for V/A-type ATPase were found in the archaeal MAGs and in the bacterial

MAGs in GAL15. Genes for F-type ATPase were found in other bacterial MAGs. No genes for terminal oxygen reductases and ATP synthase were found in the "*Nitrosocaldus*" MAGs maybe because of the low values of genome completeness.

Supplementary references

1. **Anantharaman, K., C.T. Brown, L.A. Hug*, et al.*** 2016. Thousands of microbial genomes shed light on interconnected biogeochemical processes in an aquifer system. Nat. Commun. **7:**13219.
2. **Bankevich, A., S. Nurk, D. Antipov*, et al.*** 2012. SPAdes: A new genome assembly algorithm and its applications to single-cell sequencing. J. Comput. Biol. **19:**455-477.
3. **Beam, J.P., Z.J. Jay, M.A. Kozubal, and W.P. Inskeep.** 2014. Niche specialization of novel thaumarchaeota to oxic and hypoxic acidic geothermal springs of Yellowstone National Park. ISME J. **8:**938-951.
4. **Capella-Gutierrez, S., J.M. Silla-Martinez, and T. Gabaldon.** 2009. trimAl: A tool for automated alignment trimming in large-scale phylogenetic analyses. Bioinformatics **25:**1972-1973.
5. **Edgar, R.C.** 2004. MUSCLE: Multiple sequence alignment with high accuracy and high throughput. Nucleic Acids Res. **32:**1792-1797.
6. **Hedlund, B.P., S.K. Murugapiran, T.W. Alba, A. Levy, J.A. Dodsworth, G.B. Goertz, N. Ivanova, and T. Woyke.** 2015. Uncultivated thermophiles: Current status and spotlight on 'Aigarchaeota'. Curr. Opin. Microbiol. **25:**136-145.
7. **Hirai, M., S. Nishi, M. Tsuda, M. Sunamura, Y. Takaki, and T. Nunoura.** in press. Library construction from subnanogram DNA for pelagic sea water and deep-sea sediment. Microbes Environ.
8. **Hiras, J., Y.-W. Wu, S.A. Eichorst, B.A. Simmons, and S.W. Singer.** 2016. Refining the phylum Chlorobi by resolving the phylogeny and metabolic potential of the representative of a deeply branching, uncultivated lineage. ISME J. **10:**833-845.
9. **Hug, L.A., B.C. Thomas, I. Sharon*, et al.*** 2016. Critical biogeochemical functions in the subsurface are associated with bacteria from new phyla and little studied lineages. Environ. Microbiol. **18:**159-173.
10. **Hugenholtz, P., C. Pitulle, K.L. Hershberger, and N.R. Pace.** 1998. Novel division level bacterial diversity in a Yellowstone hot spring. J. Bacteriol. **180:**366-376.
11. **Kang, D.D., J. Froula, R. Egan, and Z. Wang.** 2015. MetaBAT, an efficient tool for accurately reconstructing single genomes from complex microbial communities. PeerJ **3:**e1165.
12. **Kerou, M., P. Offre, L. Valledor, S.S. Abby, M. Melcher, M. Nagler, W. Weckwerth, and C. Schleper.** 2016. Proteomics and comparative genomics of nitrososphaera viennensis reveal the core genome and adaptations of archaeal ammonia oxidizers. Proc. Natl. Acad. Sci. USA **113:**E7937-E7946.
13. **Letunic, I., and P. Bork.** 2016. Interactive tree of life (iTOL) v3: An online tool for the display and annotation of phylogenetic and other trees. Nucleic Acids Res. **44:**W242-245.
14. **Lin, X., K.M. Handley, J.A. Gilbert, and J.E. Kostka.** 2015. Metabolic potential of fatty acid oxidation and

anaerobic respiration by abundant members of Thaumarchaeota and Thermoplasmata in deep anoxic peat. ISME J. **9:**2740-2744.

15. **Ludwig, W., O. Strunk, R. Westram*, et al.*** 2004. ARB: A software environment for sequence data. Nucleic Acids Res. **32:**1363-1371.
16. **Nishizawa, M., S. Sakai, U. Konno*, et al.*** 2016. Nitrogen and oxygen isotope effects of ammonia oxidation by thermophilic thaumarchaeota from a geothermal water stream. Appl. Environ. Microbiol. **82:**4492-4504.
17. **Ogata, H., S. Goto, K. Sato, W. Fujibuchi, H. Bono, and M. Kanehisa.** 1999. KEGG: Kyoto encyclopedia of genes and genomes. Nucleic Acids Res **27:**29-34.
18. **Parks, D.H., M. Imelfort, C.T. Skennerton, P. Hugenholtz, and G.W. Tyson.** 2015. CheckM: Assessing the quality of microbial genomes recovered from isolates, single cells, and metagenomes. Genome Res. **25:**1043-1055.
19. **Pruesse, E., J. Peplies, and F.O. Glockner.** 2012. SINA: Accurate high-throughput multiple sequence alignment of ribosomal RNA genes. Bioinformatics **28:**1823-1829.
20. **Quast, C., E. Pruesse, P. Yilmaz, J. Gerken, T. Schweer, P. Yarza, J. Peplies, and F.O. Glockner.** 2013. The SILVA ribosomal RNA gene database project: Improved data processing and web-based tools. Nucleic Acids Res. **41:**D590-596.
21. **Rinke, C., P. Schwientek, A. Sczyrba*, et al.*** 2013. Insights into the phylogeny and coding potential of microbial dark matter. Nature **499:**431-437.
22. **Seah, B.K.B., and H.R. Gruber-Vodicka.** 2015. gbtools: Interactive visualization of metagenome bins in R. Front. Microbiol. **6:**doi:10.3389/fmicb.2015.01451.
23. **Seemann, T.** 2014. Prokka: Rapid prokaryotic genome annotation. Bioinformatics **30:**2068-2069.
24. **Stamatakis, A.** 2014. RAxML version 8: A tool for phylogenetic analysis and post-analysis of large phylogenies. Bioinformatics **30:**1312-1313.
25. **Takai, K., and K. Horikoshi.** 1999. Genetic diversity of archaea in deep-sea hydrothermal vent environments. Genetics **152:**1285-1297.
26. **Takami, H., H. Noguchi, Y. Takaki*, et al.*** 2012. A deeply branching thermophilic bacterium with an ancient acetyl-CoA pathway dominates a subsurface ecosystem. PLoS ONE **7:**e30559.
27. **Takami, H., T. Taniguchi, W. Arai, K. Takemoto, Y. Moriya, and S. Goto.** 2016. An automated system for evaluation of the potential functionome: MAPLE version 2.1.0. DNA Res. **23**:467–475.
28. **Walker, C.B., J.R. de la Torre, M.G. Klotz*, et al.*** 2010. Nitrosopumilus maritimus genome reveals unique mechanisms for nitrification and autotrophy in globally distributed marine crenarchaea. Proc. Natl. Acad. Sci. USA **107:**8818-8823.
29. **Zdobnov, E.M., and R. Apweiler.** 2001. InterProScan--an integration platform for the signature-recognition methods in interpro. Bioinformatics **17:**847-848.

Supplementary figure legends

Fig. S1. Plot of G+C content vs. read coverage for the contigs of the MAGs and unbinned contigs from each sample. Bubble size indicates the length of the contigs. Colored bubbles indicate the contigs binned to MAGs.

Fig. S2. Phylogenetic tree for 16S rRNA genes. Locus tags of the MAGs obtained in this study are shown in bold. Bootstrap values (>50%) are shown at branching points. The tree was rooted at the midpoint between *Archaea* and *Bacteria.* The scale bar represents 0.1 nucleotide substitutions per sequence position.

Fig. S3. Concentrations of ammonia, nitrite, and nitrate in the influent and effluent for each bioreactor at each time point.

Fig. S4. Phylogenetic tree for NxrB/NarH proteins. Locus tags of the MAGs obtained in this study are shown in bold. Bootstrap values (>50%) are shown at branching points. The scale bar represents 0.3 amino acid substitutions per sequence position.

Fig. S5. Phylogenetic tree for Cyc2 proteins. Locus tags of the MAGs obtained in this study are shown in bold. Bootstrap values (>50%) are shown at branching points. Filled circles indicate iron-oxidizing bacteria. The scale bar represents 0.3 amino acid substitutions per sequence position.

Supplementary table legends

Table S1. Summary of sequencing results.

Table S2. Summary of sequence information for MAGs obtained in this study.

Table S3. List of the MAG metabolic potential for carbon, sulfur, and nitrogen metabolism and energy acquisition based on the detected genes.

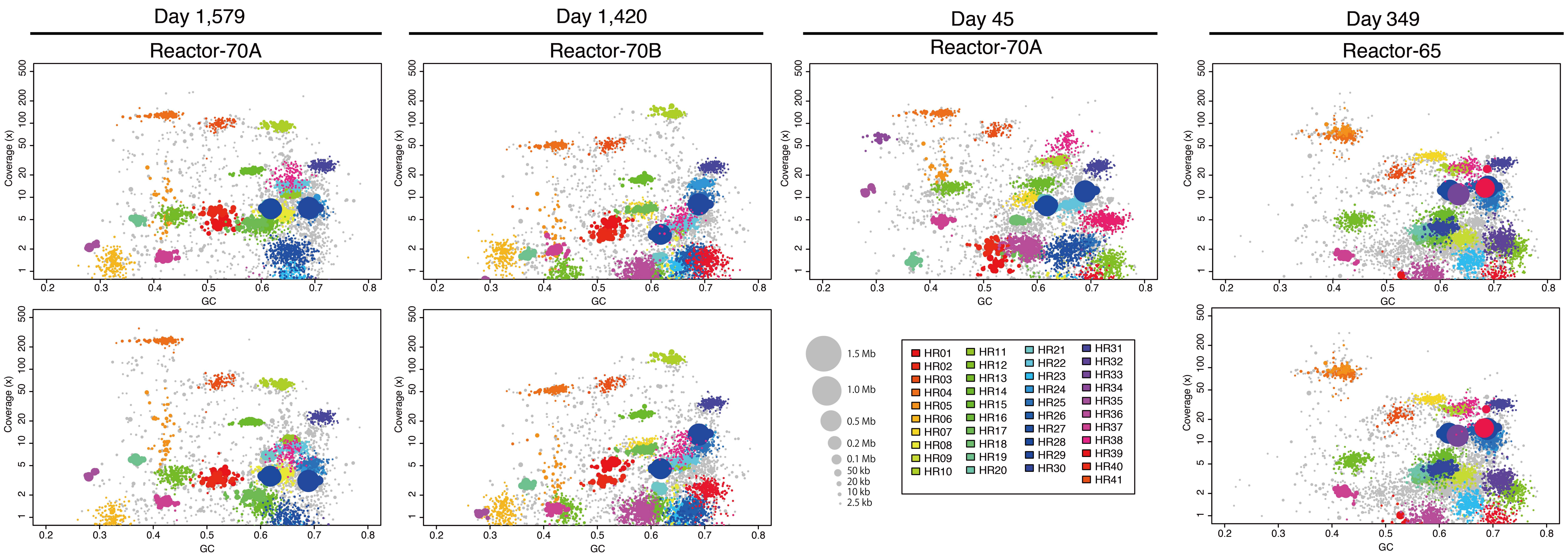
Day 1,579
Reactor-70A
Day 1,420
Reactor-70B
Day 45
Reactor-70A
Day 349
Reactor-65
Coverage (x)
GC
1.5 Mb
1.0 Mb
0.5 Mb
0.2 Mb
0.1 Mb
50 kb
20 kb
10 kb
2.5 kb
HR01
HR02
HR03
HR04
HR05
HR06
HR07
HR08
HR09
HR10
HR11
HR12
HR13
HR14
HR15
HR16
HR17
HR18
HR19
HR20
HR21
HR22
HR23
HR24
HR25
HR26
HR27
HR28
HR29
HR30
HR31
HR32
HR33
HR34
HR35
HR36
HR37
HR38
HR39
HR40
HR41

Fig. S1

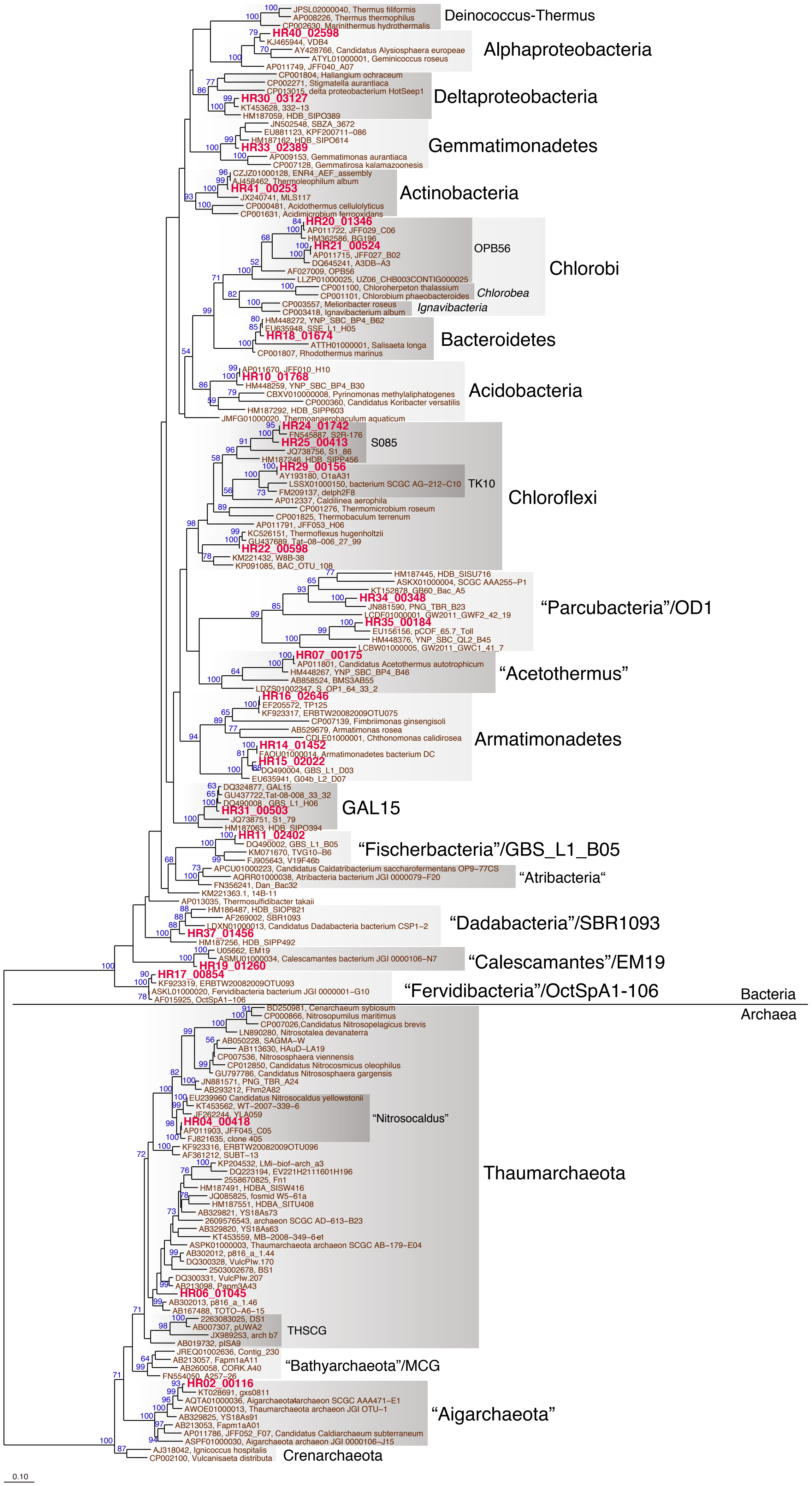

Deinococcus-Thermus
Alphaproteobacteria
Deltaproteobacteria
Gemmatimonadetes
Actinobacteria
OPB56
Chlorobi
Chlorobea
Ignavibacteria
Bacteroidetes
Acidobacteria
S085
TK10
Chloroflexi
“Parcubacteria”/OD1
“Acetothermus”
Armatimonadetes
GAL15
“Fischerbacteria”/GBS_L1_B05
“Atribacteria“
“Dadabacteria”/SBR1093
“Calescamantes”/EM19
“Fervidibacteria”/OctSpA1-106
Bacteria
Archaea
“Nitrosocaldus”
Thaumarchaeota
THSCG
“Bathyarchaeota”/MCG
“Aigarchaeota”
Crenarchaeota
HR40_02598
HR30_03127
HR33_02389
HR41_00253
HR20_01346
HR21_00524
HR18_01674
HR10_01768
HR24_01742
HR25_00413
HR29_00156
HR22_00598
HR34_00348
HR35_00184
HR07_00175
HR16_02646
HR14_01452
HR15_02022
HR31_00503
HR11_02402
HR37_01456
HR19_01260
HR17_00854
HR04_00418
HR06_01045
HR02_00116
0.10

Fig. S2

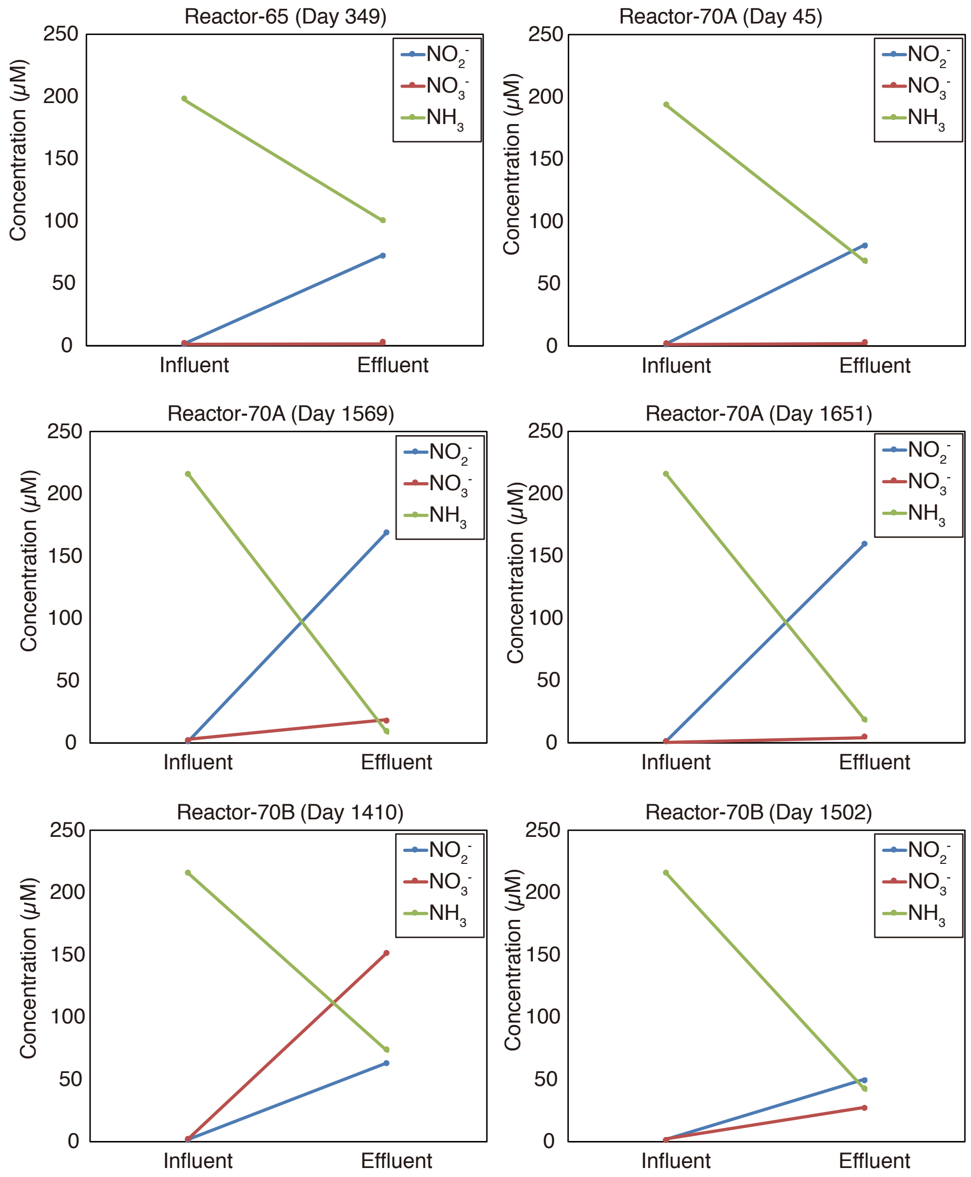
Reactor-65 (Day 349)
Reactor-70A (Day 45)
Reactor-70A (Day 1569)
Reactor-70A (Day 1651)
Reactor-70B (Day 1410)
Reactor-70B (Day 1502)
Concentration (μM)
0
50
100
150
200
250
Influent
Effluent
NO_2^-
NO_3^-
NH_3

Fig. S3

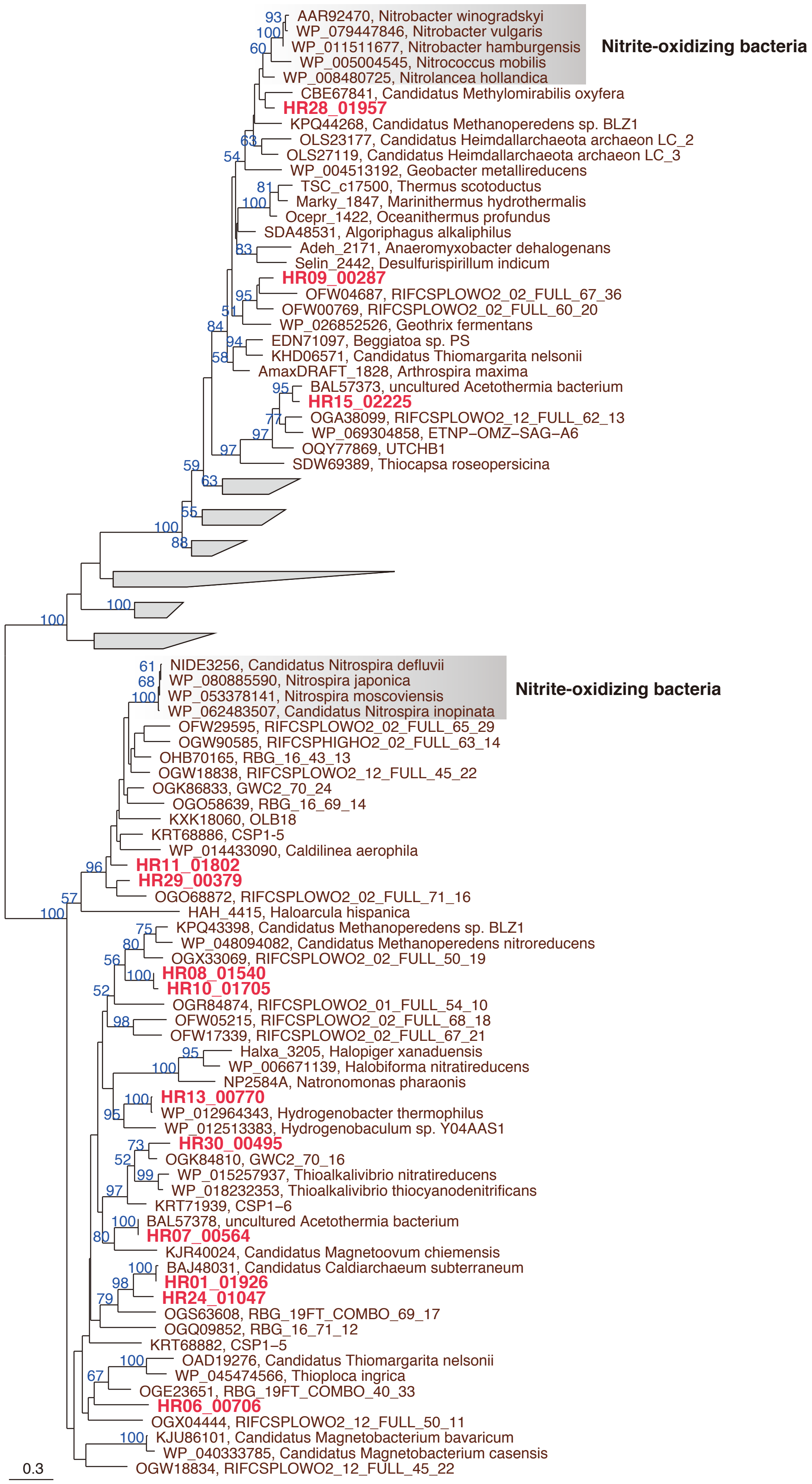
AAR92470, Nitrobacter winogradskyi
WP_079447846, Nitrobacter vulgaris
WP_011511677, Nitrobacter hamburgensis
WP_005004545, Nitrococcus mobilis
WP_008480725, Nitrolancea hollandica
Nitrite-oxidizing bacteria
CBE67841, Candidatus Methylomirabilis oxyfera
HR28_01957
KPQ44268, Candidatus Methanoperedens sp. BLZ1
OLS23177, Candidatus Heimdallarchaeota archaeon LC_2
OLS27119, Candidatus Heimdallarchaeota archaeon LC_3
WP_004513192, Geobacter metallireducens
TSC_c17500, Thermus scotoductus
Marky_1847, Marinithermus hydrothermalis
Ocepr_1422, Oceanithermus profundus
SDA48531, Algoriphagus alkaliphilus
Adeh_2171, Anaeromyxobacter dehalogenans
Selin_2442, Desulfurispirillum indicum
HR09_00287
OFW04687, RIFCSPLOWO2_02_FULL_67_36
OFW00769, RIFCSPLOWO2_02_FULL_60_20
WP_026852526, Geothrix fermentans
EDN71097, Beggiatoa sp. PS
KHD06571, Candidatus Thiomargarita nelsonii
AmaxDRAFT_1828, Arthrospira maxima
BAL57373, uncultured Acetothermia bacterium
HR15_02225
OGA38099, RIFCSPLOWO2_12_FULL_62_13
WP_069304858, ETNP-OMZ-SAG-A6
OQY77869, UTCHB1
SDW69389, Thiocapsa roseopersicina
NIDE3256, Candidatus Nitrospira defluvii
WP_080885590, Nitrospira japonica
WP_053378141, Nitrospira moscoviensis
WP_062483507, Candidatus Nitrospira inopinata
Nitrite-oxidizing bacteria
OFW29595, RIFCSPLOWO2_02_FULL_65_29
OGW90585, RIFCSPHIGHO2_02_FULL_63_14
OHB70165, RBG_16_43_13
OGW18838, RIFCSPLOWO2_12_FULL_45_22
OGK86833, GWC2_70_24
OGO58639, RBG_16_69_14
KXK18060, OLB18
KRT68886, CSP1-5
WP_014433090, Caldilinea aerophila
HR11_01802
HR29_00379
OGO68872, RIFCSPLOWO2_02_FULL_71_16
HAH_4415, Haloarcula hispanica
KPQ43398, Candidatus Methanoperedens sp. BLZ1
WP_048094082, Candidatus Methanoperedens nitroreducens
OGX33069, RIFCSPLOWO2_02_FULL_50_19
HR08_01540
HR10_01705
OGR84874, RIFCSPLOWO2_01_FULL_54_10
OFW05215, RIFCSPLOWO2_02_FULL_68_18
OFW17339, RIFCSPLOWO2_02_FULL_67_21
Halxa_3205, Halopiger xanaduensis
WP_006671139, Halobiforma nitratireducens
NP2584A, Natronomonas pharaonis
HR13_00770
WP_012964343, Hydrogenobacter thermophilus
WP_012513383, Hydrogenobaculum sp. Y04AAS1
HR30_00495
OGK84810, GWC2_70_16
WP_015257937, Thioalkalivibrio nitratireducens
WP_018232353, Thioalkalivibrio thiocyanodenitrificans
KRT71939, CSP1-6
BAL57378, uncultured Acetothermia bacterium
HR07_00564
KJR40024, Candidatus Magnetoovum chiemensis
BAJ48031, Candidatus Caldiarchaeum subterraneum
HR01_01926
HR24_01047
OGS63608, RBG_19FT_COMBO_69_17
OGQ09852, RBG_16_71_12
KRT68882, CSP1-5
OAD19276, Candidatus Thiomargarita nelsonii
WP_045474566, Thioploca ingrica
OGE23651, RBG_19FT_COMBO_40_33
HR06_00706
OGX04444, RIFCSPLOWO2_12_FULL_50_11
KJU86101, Candidatus Magnetobacterium bavaricum
WP_040333785, Candidatus Magnetobacterium casensis
OGW18834, RIFCSPLOWO2_12_FULL_45_22
0.3

Fig. S4

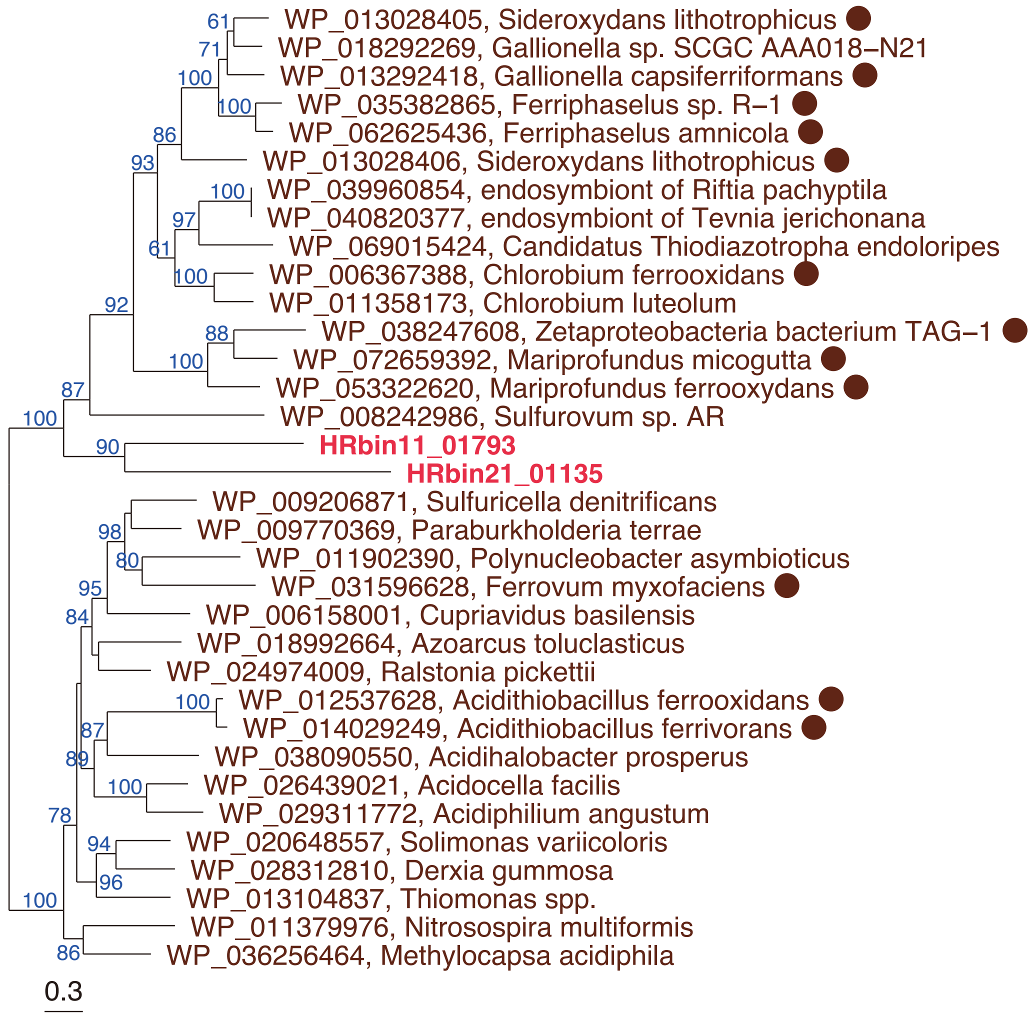
WP_013028405, Sideroxydans lithotrophicus
WP_018292269, Gallionella sp. SCGC AAA018-N21
WP_013292418, Gallionella capsiferriformans
WP_035382865, Ferriphaselus sp. R-1
WP_062625436, Ferriphaselus amnicola
WP_013028406, Sideroxydans lithotrophicus
WP_039960854, endosymbiont of Riftia pachyptila
WP_040820377, endosymbiont of Tevnia jerichonana
WP_069015424, Candidatus Thiodiazotropha endoloripes
WP_006367388, Chlorobium ferrooxidans
WP_011358173, Chlorobium luteolum
WP_038247608, Zetaproteobacteria bacterium TAG-1
WP_072659392, Mariprofundus micogutta
WP_053322620, Mariprofundus ferrooxydans
WP_008242986, Sulfurovum sp. AR
HRbin11_01793
HRbin21_01135
WP_009206871, Sulfuricella denitrificans
WP_009770369, Paraburkholderia terrae
WP_011902390, Polynucleobacter asymbioticus
WP_031596628, Ferrovum myxofaciens
WP_006158001, Cupriavidus basilensis
WP_018992664, Azoarcus toluclasticus
WP_024974009, Ralstonia pickettii
WP_012537628, Acidithiobacillus ferrooxidans
WP_014029249, Acidithiobacillus ferrivorans
WP_038090550, Acidihalobacter prosperus
WP_026439021, Acidocella facilis
WP_029311772, Acidiphilium angustum
WP_020648557, Solimonas variicoloris
WP_028312810, Derxia gummosa
WP_013104837, Thiomonas spp.
WP_011379976, Nitrosospira multiformis
WP_036256464, Methylocapsa acidiphila
0.3

Fig. S5

Table S1. Summary of sequencing results

Sample ID	DNA concentration (ug/ g sample)	Number of reads after filtering	Average length after triming (bp)	Total length (Gbp)
Reactor-70A (Day 1579)	3.10	4,669,889	286.5	1.34
	8.09	4,213,408	287.5	1.21
Reactor-70B (Day 1420)	1.04	3,925,906	284.4	1.12
	0.57	4,330,181	284.7	1.23
Reactor-70A (Day 45)	4.99	4,452,911	287.2	1.28
Reactor-65 (Day 349)	0.51	3,723,441	285.8	1.06
	0.64	4,190,286	286.3	1.20

Table S2. Summary of sequence information for MAGs obtained in this study

MAG Id	Taxa	#single-copy marker genes*				Completeness (%)	Contamination (%)	MAG size (bp)	Estimated whole-genome size (Mbp)**	Number of rRNAs	Number of tRNAs	Number of predicted CDSs	Number of contigs	Longest contig (bp)	N50 (bp)	Mean contig length (bp)	GC	Average read coverage (x)							Accession number
																		Day 1,579		Day 1,420		Day 45	Day 349		
		0	1	2	3+													Reactor-70A (in duplicate)		Reactor-70B (in duplicate)		Reactor-70A	Reactor-65 (in duplicate)		
HR01	Aigarchaeota	3	142	0	0	97.1	0	1761185	1.81	23S (partial) x2, 5S x1	28	1916	32	180679	96642	55037	52.6%	4.44	3.55	4.42	5.29	1.41	0.58	0.65	BEHD01000001-BEHD01000032
HR02	Aigarchaeota	2	143	0	0	98.1	0	1733307	1.77	16S (partial) x1, 23S (partial) x1, 5S x1	31	1939	36	155245	84639	48147	51.4%	6.50	3.41	3.30	3.33	2.33	0.11	0.10	BEHE01000001-BEHE01000036
HR03	Aigarchaeota	103	42	0	0	27.6	0	393979	1.43	-	7	434	92	20513	4031	4282	52.3%	97.43	67.69	51.17	63.49	78.89	21.42	22.76	BEHF01000001-BEHF01000092
HR04	Nitrosocaldus	38	106	1	0	76.5	0.32	1208457	1.58	16S (partial) x1	19	1362	142	43290	10746	8510	41.8%	127.91	242.28	49.39	52.32	135.90	67.60	83.78	BEHG01000001-BEHG01000142
HR05	Nitrosocaldus	62	83	0	0	58.5	0	635432	1.09	-	18	721	45	46676	17383	14121	41.8%	9.17	17.01	4.00	4.28	24.21	80.88	95.15	BEHH01000001-BEHH01000045
HR06	Papm3A43-related	57	87	1	0	61.0	0.97	992123	1.63	16S (partial) x1, 5S x1	14	1119	213	16387	4752	4658	32.4%	1.27	0.86	1.73	1.23	0.01	0.00	0.00	BEHI01000001-BEHI01000213
HR07	Acetothermia	35	69	0	0	54.2	0	685016	1.26	16S x1	13	726	149	14844	4877	4597	58.5%	0.14	0.07	6.83	9.36	9.67	35.89	37.63	BEHJ01000001-BEHJ01000149
HR08	Acidobacteria	40	63	1	0	73.3	1.72	2602175	3.55	-	35	2256	197	51853	19655	13209	63.7%	5.84	3.81	0.57	0.76	0.28	0.06	0.08	BEHK01000001-BEHK01000197
HR09	Acidobacteria	8	94	2	0	88.8	2.59	2347125	2.64	-	44	2103	309	28749	8952	7596	63.1%	0.00	0.00	0.00	0.00	0.08	2.82	3.52	BEHL01000001-BEHL01000309
HR10	Acidobacteria	7	94	3	0	94.2	2.74	3131958	3.32	16S x1	46	2687	118	219437	65038	26542	63.8%	90.21	62.40	134.11	136.06	31.31	23.99	27.21	BEHM01000001-BEHM01000118
HR11	Fischerbacteria/GBS-L1-B05	1	103	0	0	98.3	0	2988257	3.04	16S x1, 23S (partial) x1, 5S x1	48	2436	49	397756	105948	60985	65.7%	10.99	10.85	3.92	5.10	0.50	0.01	0.00	BEHN01000001-BEHN01000049
HR12	Actinobacteria	29	73	2	0	68.7	1.72	1762032	2.57	-	40	1868	361	23389	5185	4881	73.3%	0.00	0.00	0.00	0.00	1.13	2.09	2.13	BEHO01000001-BEHO01000361
HR13	Aquificae	17	87	0	0	82.7	0	1337495	1.62	16S x1, 23S (partial) x1, 5S x1	30	1391	187	29049	7960	7152	44.1%	5.73	3.70	0.92	1.23	13.64	4.99	5.57	BEHP01000001-BEHP01000187
HR14	Armatimonadetes	10	93	1	0	84.5	0.16	2515047	2.98	16S x1, 23S x1, 5S x1	49	2268	265	64527	13400	9491	60.7%	4.14	1.47	0.69	1.36	15.27	5.64	6.16	BEHQ01000001-BEHQ01000265
HR15	Armatimonadetes	2	102	0	0	97.4	0	3036312	3.12	16S (partial) x1, 23S x1, 5S x1	52	2672	72	202200	68036	42171	58.1%	22.76	19.27	17.19	24.69	0.35	0.03	0.03	BEHR01000001-BEHR01000072
HR16	Armatimonadetes	12	92	0	0	89.6	0	3785506	4.22	16S x1, 23S x1, 5S x1	40	3294	325	81076	15427	11648	59.2%	0.06	0.02	0.07	0.08	0.34	3.24	3.74	BEHS01000001-BEHS01000325
HR17	Fervidibacteria/Oct-Spa1-106	4	100	0	0	93.1	0	3153697	3.39	16S (partial) x1	51	2781	75	172999	69561	42049	58.6%	4.42	2.03	6.95	8.10	1.79	0.00	0.00	BEHT01000001-BEHT01000075
HR18	Bacteroidetes	1	103	0	0	98.3	0	2853867	2.90	16S x1, 23S x1	44	2438	29	228791	156798	98409	56.3%	0.19	0.06	0.29	0.37	4.93	2.90	3.25	BEHU01000001-BEHU01000029
HR19	Calescamantes/EM19	16	88	0	0	75.9	0	1875992	2.47	16S x1	46	1704	22	262593	124983	85272	36.9%	4.98	6.02	1.68	2.74	1.36	0.01	0.00	BEHV01000001-BEHV01000022
HR20	Chlorobi	13	91	0	0	91.9	0	1993452	2.17	16S x1, 23S x1, 5S x1	39	1779	147	50341	18151	13561	57.3%	0.00	0.00	0.00	0.00	0.03	3.57	3.92	BEHW01000001-BEHW01000147
HR21	Chlorobi	4	100	0	0	93.1	0	1834294	1.97	16S x1, 23S x1, 5S x1	47	1558	17	350506	177521	107900	61.7%	7.89	6.50	1.51	2.48	9.40	0.01	0.01	BEHX01000001-BEHX01000017
HR22	Chloroflexi	4	99	1	0	94.8	0.34	2931246	3.09	5S x1	43	2585	175	127152	34479	16750	66.1%	14.64	8.59	4.47	5.91	7.92	0.02	0.01	BEHY01000001-BEHY01000175
HR23	Chloroflexi	18	86	0	0	79.8	0	1621116	2.03	16S x1, 23S (partial) x1, 5S x1	44	1642	175	34979	12467	9264	65.6%	0.00	0.00	0.00	0.00	1.13	2.09	2.13	BEHZ01000001-BEHZ01000175
HR24	Chloroflexi	4	100	0	0	95.5	0	2051769	2.15	16S x1	49	2131	159	137298	17808	12904	69.3%	7.79	4.64	15.10	12.97	0.43	10.49	11.64	BEIA01000001-BEIA01000159
HR25	Chloroflexi	59	45	0	0	60.7	0	989871	1.63	-	23	982	106	74878	13954	9338	69.5%	6.24	5.29	10.39	8.69	2.33	8.77	9.88	BEIB01000001-BEIB01000106
HR26	Chloroflexi	14	90	0	0	79.3	0	2664985	3.36	-	38	2404	319	44900	10628	8354	67.9%	0.36	0.15	1.48	1.25	0.31	14.17	14.74	BEIC01000001-BEIC01000319
HR27	Chloroflexi	43	60	1	0	66.3	1.72	2073354	3.13	23S (partial) x1	25	2050	419	18001	5290	4948	65.3%	1.75	0.82	0.37	0.54	1.91	0.04	0.03	BEID01000001-BEID01000419
HR28	Chloroflexi	1	103	0	0	98.3	0	3123359	3.18	16S x1, 23S x1, 5S x1	52	2802	8	1387553	754814	390420	61.6%	7.05	3.53	3.08	4.40	7.68	12.33	12.76	BEIE01000001-BEIE01000008
HR29	Chloroflexi	3	101	0	0	95.7	0	2343573	2.45	16S (partial) x1	49	2317	4	1557145	2E+06	585893	69.3%	7.10	3.15	8.00	13.32	12.28	13.97	15.16	BEIF01000001-BEIF01000004
HR30	Deltaproteobacteria	4	100	0	0	94.5	0	3727088	3.94	-	44	3302	211	91819	28735	17664	60.3%	0.00	0.00	0.00	0.00	0.01	3.87	4.34	BEIG01000001-BEIG01000211
HR31	GAL15	38	66	0	0	65.7	0	945340	1.44	16S x1, 23S (partial) x1	14	927	227	12021	4171	4164	71.3%	26.89	22.64	25.93	35.05	26.24	29.02	32.17	BEIH01000001-BEIH01000226
HR32	GAL15	33	71	0	0	65.8	0	2102379	3.19	-	33	2139	380	27788	6114	5533	71.3%	0.03	0.01	0.04	0.06	0.04	2.66	3.03	BEII01000001-BEII01000380
HR33	Gemmatimonas	0	104	0	0	100.0	0	2944810	2.94	16S x1, 23S x1, 5S x1	50	2662	5	1549729	2E+06	588962	63.4%	0.00	0.00	0.01	0.01	0.01	10.67	11.85	BEIJ01000001-BEIJ01000005
HR34	Parcubacteria/OD1	13	91	0	0	79.6	0	579108	0.73	16S x1, 23S x1, 5S x1	45	573	22	87454	53001	26323	30.7%	0.01	0.01	0.03	0.01	62.97	0.01	0.01	BEIK01000001-BEIK01000022
HR35	Parcubacteria/OD1	16	88	0	0	73.6	0	595782	0.81	16S x1, 23S x1	45	609	5	247965	152089	119156	28.5%	2.22	3.93	0.72	1.14	12.54	0.01	0.01	BEIL01000001-BEIL01000005
HR36	Planctomyses	15	89	0	0	86.1	0	3355873	3.90	23S x1, 5S x1	33	2863	467	32917	8395	7186	57.9%	0.32	0.16	0.96	1.20	2.16	0.75	0.80	BEIM01000001-BEIM01000467
HR37	Dadabacteria/SBR1093	1	102	1	0	98.3	1.72	2420114	2.46	16S x1, 23S x1, 5S x1	48	2384	33	348728	100452	73337	42.2%	1.60	1.58	1.94	1.22	4.90	1.62	2.04	BEIN01000001-BEIN01000033
HR38	Deinococcus-Thermus	94	10	0	0	16.4	0	458133	2.80	-	9	485	129	7939	3480	3551	65.2%	19.57	7.56	5.10	9.02	53.46	24.98	28.53	BEIO01000001-BEIO01000129
HR39	Alphaproteobacteria	24	80	0	0	74.8	0	1992583	2.67	-	25	1916	396	23613	5294	5032	72.6%	0.02	0.01	0.31	0.28	4.90	0.02	0.02	BEIP01000001-BEIP01000396
HR40	Alphaproteobacteria	1	103	0	0	98.3	0	2892134	2.94	16S (partial) x1	48	2654	8	1180418	611759	361517	68.6%	0.00	0.00	0.01	0.00	0.11	14.89	16.82	BEIQ01000001-BEIQ01000008
HR41	Actinobacteria	30	74	0	0	65.5	0	1610524	2.46	16S x1, 23S x1, 5S x1	33	1586	285	26672	6277	5651	70.5%	0.12	0.02	1.36	2.32	0.58	0.62	0.70	BEIR01000001-BEIR01000285

*a total of 145 and 104 single-copy maker genes for the domains *Archaea* and *Bacteria*; ** calculated based on the genome completeness and MAG size

Table S3. List of the MAG metabolic potential for carbon, sulfur, and nitrogen metabolism and energy acquisition based on the detected genes

Metabolism* — Carbon fixation

		Calvin cycle		Reductive TCA cycle		Reductive acetyl-CoA pathway		Dicarboxylate/4HB cycle; 3HP/4HB cycle; 3HP bicycle					
MAG ID	Taxa	M00165	rbcSL	M00173	acl/ccs /ccl	M00377	coo/fdh	M00374	M00375	M00376	accAB C	pccAB	abfD
HR01	Aigarchaeota	27.3		81.8				46.2					
HR02	Aigarchaeota	27.3		90.9				53.8					
HR03	Aigarchaeota			27.3									
HR04	Nitrosocaldus	45.5		45.5				38.5	28.6		accA		abfD
HR05	Nitrosocaldus			50									
HR06	Papm3A43/TOTO			54.5				30.8					
HR07	Acetothermia			30									
HR08	Acidobacteria	36.4		72.7				38.5		30.8			
HR09	Acidobacteria	36.4		72.7		28.6		46.2			accAC		
HR10	Acidobacteria	45.5		72.7		28.6		38.5		30.8			
HR11	Fischerbacteria	72.7	rbcSL	72.7		28.6		38.5		30.8			
HR12	Actinobacteria	27.3		63.6		42.9		46.2		30.8			
HR13	Aquificae	27.3		72.7	ccl			30.8					
HR14	Armatimonadetes	45.5				28.6							
HR15	Armatimonadetes	54.5		54.5		28.6				30.8			
HR16	Armatimonadetes	63.6		81.8		42.9		30.8					
HR17	Fervidibacteria	45.5		81.8		28.6		30.8					
HR18	Bacteroidetes	63.6	rbcL	81.8		28.6		38.5					
HR19	Calescamantes	36.4		72.7		28.6		30.8		30.8	accC		
HR20	Chlorobi	54.5		40		28.6				30.8			
HR21	Chlorobi	36.4		50		28.6							
HR22	Chloroflexi	54.5		81.8				30.8		30.8			
HR23	Chloroflexi	36.4		81.8		42.9		46.2		30.8			
HR24	Chloroflexi	45.5		60		28.6		30.8		30.8			
HR25	Chloroflexi	27.3		60									
HR26	Chloroflexi	54.5		63.6		42.9		46.2		30.8			
HR27	Chloroflexi	45.5		45.5		42.9		30.8					
HR28	Chloroflexi	54.5		72.7		42.9		46.2		38.5			
HR29	Chloroflexi	54.5		72.7				46.2		38.5	accABC		
HR30	Deltaproteobacteria	45.5		81.8		42.9		38.5		38.5			
HR31	GAL15	45.5		60				38.5		30.8	accAB		
HR32	GAL15	36.4		72.7		28.6		46.2					
HR33	Gemmatimonas	63.6	rbcL	72.7		42.9		30.8	28.6	30.8	accABC		
HR34	Parcubacteria	63.6											
HR35	Parcubacteria	63.6											
HR36	Planctomyses	36.4		90				30.8					
HR37	Dadabacteria	45.5		90.9		28.6		46.2					
HR38	Deinococcus-Thermus	27.3		27.3									
HR39	Alphaproteobacteria	54.5	rbcL	72.7		28.6		53.8		46.2			
HR40	Alphaproteobacteria	90.9	rbcSL	81.8		42.9		53.8		61.5			
HR41	Actinobacteria	54.5		60		28.6		30.8		30.8			

Metabolism* — Nitrogen metabolism; Sulfur metabolism

		Nitrogen fixation	Assimilatory nitrate reduction		Dissimilatory nitrate reduction		Denitrification			Nitrification	Nitrite oxidation	Assimilatory sulfate reduction	Thiosulfate oxidation
MAG ID	Taxa	nifDKH	narB/ nasA B	nirA	napGHI/n arAB	nirBD/ nrfAH	nirS/ nirK	norBC	nosZ	amoABC	nxrAB	M00176	M00595
HR01	Aigarchaeota				narH		nirK				nxrB	33.3	
HR02	Aigarchaeota						nirK		nosZ				
HR03	Aigarchaeota												
HR04	Nitrosocaldus									amoABC		33.3	
HR05	Nitrosocaldus									amoABC			
HR06	Papm3A43/TOTO				narGH		nirK				nxrAB	33.3	
HR07	Acetothermia				narH						nxrB		
HR08	Acidobacteria				narGH			norB			nxrAB	33.3	
HR09	Acidobacteria				narHI/narA			norBC			nxrB		
HR10	Acidobacteria				narGH			norB			nxrAB	100	
HR11	Fischerbacteria			nirA	narGH	nirD			nosZ		nxrAB	66.7	
HR12	Actinobacteria			nirA		nrfAH			nosZ			33.3	
HR13	Aquificae	nifDHK	narB		narGH	nirB	nirS	norBC	nosZ		nxrAB		
HR14	Armatimonadetes								nosZ				
HR15	Armatimonadetes				narGHI				nosZ		nxrAB		
HR16	Armatimonadetes			nirA		nrfA						66.7	
HR17	Fervidibacteria			nirA		nrfA		norB	nosZ			100	
HR18	Bacteroidetes		narB	nirA	narA		nirK		nosZ			33.3	
HR19	Calescamantes						nirS		nosZ			100	
HR20	Chlorobi								nosZ				
HR21	Chlorobi								nosZ				
HR22	Chloroflexi					nrfAH	nirK		nosZ				
HR23	Chloroflexi											33.3	
HR24	Chloroflexi				narGH		nirK				nxrAB	66.7	
HR25	Chloroflexi			nirA								33.3	
HR26	Chloroflexi								nosZ				
HR27	Chloroflexi						nirK		nosZ				
HR28	Chloroflexi				narGHI		nirK		nosZ		nxrAB		
HR29	Chloroflexi			nirA	narGH		nirK		nosZ		nxrAB	100	
HR30	Deltaproteobacteria		nasA		narGH	nirBD			nosZ		nxrAB	66.7	
HR31	GAL15												
HR32	GAL15			nirA			nirK	norB				66.7	
HR33	Gemmatimonas				narA				nosZ				
HR34	Parcubacteria												
HR35	Parcubacteria												
HR36	Planctomyses			nirA		nrfAH		norB				33.3	
HR37	Dadabacteria			nirA					nosZ			66.7	
HR38	Deinococcus-Thermus												
HR39	Alphaproteobacteria						nirS					66.7	100
HR40	Alphaproteobacteria						nirS					66.7	
HR41	Actinobacteria		narB				nirK					66.7	

Metabolism* — Central carbohydrate metabolism; Fatty acid metabolism; Fermentation; Aerobic respirasion; ATP synthesis

		EM pathway	Gluconeo genesis	ED pathway	Pyruvate oxidation	TCA cycle	acyl-CoA synthesis	beta-Oxidation	Acetate production	Lactate <-> Pyruvate	Alcohol <-> Aldehydes	Cyt c oxidase	Cyt d ubiquinol oxidase	Cyt c oxidase, cbb3-type	F-type ATPase	V/A-type ATPase
MAG ID	Taxa	M00001	M00003	M00008	M00307	M00009	M00086	M00087	M00579	LDH	ADH	M00155	M00153	M00156	M00157	M00159
HR01	Aigarchaeota	90	85.7		100	87.5	100	50				66.7				77.8
HR02	Aigarchaeota	80	85.7		100	87.5	100	33.3			ADH	66.7	50			55.6
HR03	Aigarchaeota		28.6			25		25				33.3				44.4
HR04	Nitrosocaldus	40	85.7			37.5					ADH					
HR05	Nitrosocaldus	30	42.9			62.5					ADH					
HR06	Papm3A43/TOTO	40	37.5			50	100				ADH	100				44.4
HR07	Acetothermia	30	57.1			50	100				ADH	50		25		44.4
HR08	Acidobacteria	90	100	50	100	62.5	100	100				100			50	
HR09	Acidobacteria	70	75			100	100	75	100		ADH	100			62.5	
HR10	Acidobacteria	90	100	50	100	62.5	100	100				100			75	
HR11	Fischerbacteria	80	100	25	100	75	100	75			ADH	66.7		50	75	
HR12	Actinobacteria	80	85.7		100	75	100					100	50		62.5	
HR13	Aquificae	80	75		100	75	100		50			100			87.5	
HR14	Armatimonadetes	90	87.5	25			100	50		LDH	ADH	50			87.5	
HR15	Armatimonadetes	100	100		100	75	100	50		LDH	ADH	100			87.5	
HR16	Armatimonadetes	100	87.5	75	100	100	100		50	LDH		100			75	
HR17	Fervidibacteria	90	71.4	50		100	100			LDH		100			87.5	
HR18	Bacteroidetes	100	100	25	100	87.5	100	75		LDH	ADH	100			100	
HR19	Calescamantes	70	100			87.5	100	33.3				100			62.5	
HR20	Chlorobi	100	100			62.5	100	25	50			100			87.5	
HR21	Chlorobi	77.8	62.5			75	100	25	50			66.7			62.5	
HR22	Chloroflexi	90	85.7	75	100	87.5	100	100			ADH	100		33.3	75	
HR23	Chloroflexi	50	57.1		100	100		50	50		ADH	100			62.5	
HR24	Chloroflexi	80	85.7			75	100	75			ADH	100			100	
HR25	Chloroflexi		28.6			50	100	50			ADH				87.5	
HR26	Chloroflexi	80	87.5	75		87.5					ADH	66.7			100	
HR27	Chloroflexi	70	50	50		50		50			ADH	66.7			100	
HR28	Chloroflexi	90	75	75	100	87.5	100	75			ADH	66.7			100	
HR29	Chloroflexi	90	75		100	100	100	75			ADH	100	100		100	
HR30	Deltaproteobacteria	90	100	50	100	100	100	100	50		ADH	100			75	
HR31	GAL15	50	85.7			75	100	75			ADH	100				55.6
HR32	GAL15	80	100	50	100	75	100	100			ADH	50		25		55.6
HR33	Gemmatimonas	90	100		100	87.5	100	100	50		ADH	100		33.3	87.5	
HR34	Parcubacteria	80	75												62.5	
HR35	Parcubacteria	50	62.5												50	
HR36	Planctomyses	80	57.1	25	100	87.5	100	66.7	100	LDH		100			87.5	
HR37	Dadabacteria	100	75	25	100	87.5	100	100			ADH	100		75	100	
HR38	Deinococcus-Thermus	50	50		100			33.3				100				
HR39	Alphaproteobacteria	60	50		100	87.5	100	33.3				100		75	87.5	
HR40	Alphaproteobacteria	100	87.5		100	87.5	100	75			ADH	100		75	100	
HR41	Actinobacteria	80	75	50	100	75		50			ADH	100			37.5	

*the module completion ratio (MCR) determined by MAPLE (only >25% are shown) for the KEGG module ID, or the presence/absence for key enzymes are shown
